# Brain plasticity in pregnancy and the postpartum period: links to maternal caregiving and mental health

**DOI:** 10.1007/s00737-018-0889-z

**Published:** 2018-07-14

**Authors:** Erika Barba-Müller, Sinéad Craddock, Susanna Carmona, Elseline Hoekzema

**Affiliations:** 1 Brain and Development Research Center, Leiden University, Leiden, the Netherlands; 2 Leiden Institute for Brain and Cognition, Leiden, the Netherlands; 3 University Institute of Mental Health Vidal i Barraquer, Ramon Llull University, Barcelona, Spain; 4 Centro de Investigación Biomédica en Red de Salud Mental (CIBERSAM), Madrid, Spain; 5 Unidad de Medicina y Cirugía Experimental, Instituto de Investigación Sanitaria Gregorio Marañón, Madrid, Spain; 6 Faculty of Health Sciences, Universitat Oberta de Catalunya, Barcelona, Spain

**Keywords:** Neuroplasticity, Perinatal mental health, Maternal behavior, Postpartum depression, Maternal attachment

## Abstract

Pregnancy and the postpartum period involve numerous physiological adaptations that enable the development and survival of the offspring. A distinct neural plasticity characterizes the female brain during this period, and dynamic structural and functional changes take place that accompany fundamental behavioral adaptations, stimulating the female to progress from an individual with self-directed needs to being responsible for the care of another life. While many animal studies detail these modifications, an emerging body of research reveals the existence of reproduction-related brain plasticity in human mothers too. Additionally, associations with aspects of maternal caregiving point to adaptive changes that benefit a woman’s transition to motherhood. However, the dynamic changes that affect a woman’s brain are not merely adaptive, and they likely confer a vulnerability for the development of mental disorders. Here, we review the changes in brain structure and function that a woman undergoes during the peripartum period, outlining associations between these neural alterations and different aspects of maternal care. We additionally discuss peripartum mood disorders and postpartum psychosis, and review the neuroimaging studies that investigate the neural bases of these conditions.

## Introduction

Becoming a mother brings about profound changes in the female mammalian brain. Evidence from across the animal kingdom indicates numerous functional and structural adaptations in the female brain throughout pregnancy and the postpartum period, which are driven by an interplay between endocrine and environmental factors (Brunton and Russell 2008; Hillerer et al. 2014a; Kohl et al. 2016). These reproduction-related changes take place in brain structures that regulate maternal caregiving behaviors such as licking and grooming of the young, with the medial preoptic area (mPOA) as a core node in the induction of these behaviors (Keyser-Marcus et al. 2001; Numan 2007), but they also occur in structures that are not directly associated with this behavioral repertoire of caregiving activities, such as the hippocampal complex (Pawluski et al. 2016). At the cellular level, the events that take place are also wide-ranging, including various forms of neural plasticity such as neurogenesis, synaptic remodeling and changes (increases and/or decreases) in dendritic morphometry, spine density, and astrocytic density (Kinsley et al. 2006; Leuner and Sabihi 2016; Salmaso et al. 2009). The resulting maternal network comprises multiple cortical, limbic, and sensory systems that interact to support various forms of maternal behavior. Interestingly, many of the regions of the maternal circuitry are rich in receptors for oxytocin—a neuropeptide that plays a key role in bonding—and are considered central nodes of the mammalian social behavior network (Kohl and Dulac 2018; Newman 1999).

It is interesting to note that some of these structural brain changes were replicated in virgin female rodents after a hormonal treatment mimicking pregnancy based on exogenous steroid hormones, stressing the implication of the hormonal surges of pregnancy in the observed gestational brain changes (Keyser-Marcus et al. 2001; Kinsley et al. 2006). This is unsurprising, since sex steroid hormones are powerful neurotrophic agents that regulate most major developmental events (Simerly 2002). Other hormones such as prolactin, oxytocin, and glucocorticoids have also been shown to play a key role in shaping or activating the maternal brain (Kim and Strathearn 2016; Slattery and Hillerer 2016). For instance, in pregnant mice, increases in prolactin mediate the production of neural progenitors in the forebrain subventricular zone, which later migrate to produce new olfactory interneurons, likely facilitating the recognition and rearing of the offspring (Shingo et al. 2003). Once endocrine factors have triggered the neural circuits regulating maternal behavior, this behavior is maintained by experiential factors, i.e., by exposure to offspring cues (Numan 2007).

Research investigating the human brain in relation to pregnancy and the postpartum period pales in comparison to animal research in this domain. However, emerging magnetic resonance imaging (MRI) studies indicate that the human brain also undergoes dramatic changes during pregnancy and the postpartum period. Here, we review the structural and functional brain changes that take place during the gestational and postpartum period in women. First, we review the very scarce research detailing changes in a woman’s brain structure, proceeding to a more extensive discussion of the studies investigating brain function in the peripartum period. Furthermore, throughout these sections, we highlight findings that link this neuroplasticity to aspects of maternal caregiving. Finally, we introduce the main peripartum mental disorders, by briefly describing the symptoms, incidence rates, and principal risk factors associated with these conditions, followed by a discussion of the neuroimaging findings that shed light on the neurobiology of these debilitating disorders.

## Structural brain plasticity

Like other mammals, a woman experiences unparalleled surges of sex steroid hormones during pregnancy (Casey et al. 1993) and some authors have suggested the existence of a structural brain plasticity inherent and specific to human reproduction itself (Brunton and Russell 2008; Kinsley and Amory-Meyer 2011; Swain et al. 2014). While this topic has scarcely been investigated, a few studies point to the occurrence of dynamic peripartum changes in a woman’s brain structure. For instance, it was recently found that first-time mothers undergo a symmetrical pattern of extensive gray matter (GM) volume reductions across pregnancy, primarily affecting the anterior and posterior cortical midline and specific sections of the bilateral lateral prefrontal and temporal cortex, which last for at least 2 years postpartum (Hoekzema et al. 2017). The affected areas were found to significantly overlap with the Theory of Mind (ToM) network and with brain activations in response to own-baby stimuli in the postpartum period. It is noteworthy that these changes consist exclusively of volume reductions and no increases were detected. Accordingly, other studies have found a decrease in overall brain size related to pregnancy in humans (Oatridge et al. 2002) and rodents (Hillerer et al. 2014b). Moreover, GM volume reductions have been observed in response to increases in endogenous or exogenous sex steroid hormones outside of pregnancy, for instance during adolescence (Peper et al. 2011) or hormonal treatment in gender dysphoric individuals (Zubiaurre-Elorza et al. 2014), which supports the notion that hormones are the main mediators of the morphometric brain changes that accompany gestation.

Furthermore, there are indications that brain structure continues to develop in the postpartum period. Based on findings from animal studies, we can speculate that, while hormonal factors are paramount in driving neural adaptations during pregnancy, direct interactions with the baby play a key role in further postpartum brain changes. Infants’ needs are always evolving throughout development and parents intuitively adapt to those changes, incorporating new knowledge, abilities and skills in their parental repertoire. This results in a neural reorganization referred to as experience-dependent plasticity (Nithianantharajah and Hannan 2006).

Kim et al. (2010) assessed morphological changes of the mother’s brain during the postpartum period. Results revealed that from time 1 (2–4 weeks postpartum) to time 2 (3–4 months postpartum) mothers showed increases in GM volume in sections of the parietal lobe, prefrontal cortex, and midbrain. In contrast to the observed structural brain changes across pregnancy, no GM decreases were detected in the early postpartum period. Although it is difficult to directly compare these findings due to differences in study parameters (e.g., time period between scans, statistical thresholds, the number of participants, and morphometric procedures), these findings suggest that pregnancy and motherhood exert divergent—even opposing—effects on parts of a woman’s brain in terms of the direction of GM volume change. While these findings mostly concern distinct sections of the brain—potentially reflecting a region-dependent sensitivity to reproduction-related endocrine versus experiential factors—there is some overlap in the involved anatomical areas as well. These areas of overlap primarily concern clusters located along the midline of the brain, which were found to undergo increases during the early postpartum period as well as volume losses across pregnancy that remained stable for at least 2 years postpartum. This might seem contradictory but could reflect, for instance, a partial volume recovery within these regions following the strong volume reductions of pregnancy that had already (partially) occurred prior to the post-pregnancy follow-up scan.

Interestingly, a positive perception of the baby predicted greater GM augmentations in the hypothalamus, substantia nigra, and amygdala in the mothers’ brains (Kim et al. 2010), pointing to a relation between the changes in maternal brain structure and a mother’s response to her baby. Furthermore, there are indications that changes in a woman’s brain structure across pregnancy are also linked to aspects of maternal caregiving, that is, to the development of maternal attachment. The brain changes that occur during pregnancy were found to significantly predict the quality of mother-to-infant attachment and the absence of hostility towards her newborn (Hoekzema et al. 2017).

While more research on this topic is needed, these studies assessing morphometric changes draw a first impression of certain—primarily social—brain areas that undergo decreases in GM volume driven by hormone-derived plasticity in pregnancy, followed by GM volume increases—primarily in networks involved in motivation, somatosensory information and executive functions—mediated by experience-dependent plasticity during the postpartum period. Furthermore, there are indications that both gestational and postpartum changes in brain structure are linked to aspects of maternal caregiving, providing preliminary evidence for an adaptive purpose serving a woman’s transition to motherhood. Future longitudinal studies collecting hormonal samples and detailed measures of environmental changes during pregnancy and the postpartum period are necessary to elucidate the mediators contributing to the brain plasticity inherent to reproduction and to better track the structural changes in a woman’s brain across this dynamic period.

## Functional brain plasticity

In contrast to the few studies examining structural brain changes in human mothers, there are several studies that assess brain activity in response to own-infant stimuli in comparison to other-infant stimuli using functional magnetic resonance imaging (fMRI) in the postpartum period. These studies allow us to identify regions of the brain involved in motherhood by measuring activity in the mothers’ brains in response to their babies’ auditory or visual stimuli. Taken together, they contribute to our understanding of the neural substrates that underlie the expression of maternal behavior (Barrett and Fleming 2011; Kim et al. 2016; Moses-Kolko et al. 2014; Noriuchi et al. 2008; Swain et al. 2014; Young et al. 2017).

While numerous brain regions have been observed and paradigms often differ across laboratories, certain networks have been replicated across various studies that are considered part of the maternal brain. These networks are not completely independent, they share common brain regions and can be co-activated.

When mothers are presented with stimuli of their own child, areas of the *reward system* become activated. This is unsurprising, since seeing a loved one is a rewarding experience, which is even stronger during early motherhood, when her baby becomes a mother’s most powerful appetitive stimulus. Studies in female rodents show that mothers eagerly bar-press for contact with pups (Lee et al. 2000) and find pup suckling more rewarding than cocaine (Ferris 2005). Therefore, this dopaminergic and oxytocinergic neuroendocrine system is also called the *maternal motivation system*, which has been defined by animal studies. The core regions of the maternal motivational system comprise the mPOA and the bed nucleus of the stria terminalis, which project to the ventral tegmental area and the retrorubral field and activate the reward circuit by releasing dopamine into the nucleus accumbens (Numan 2007). In addition to the mesolimbic reward pathway connecting the ventral tegmental area and nucleus accumbens, many other subcortical and cortical structures contribute to the processing of rewarding experiences, forming a complex network that interfaces with cognitive functions to affect motor planning, like the dorsal striatum, substantia nigra, amygdala, hippocampus, and various prefrontal areas (Berridge and Kringelbach 2015; Haber and Knutson 2010). In human mothers, many of these brain regions have been observed to respond strongly when presented with cues of their infants (Bartels and Zeki 2004; Lorberbaum et al. 2002; Noriuchi et al. 2008), even more so if the baby is smiling (Strathearn et al. 2008).

Another conclusion from fMRI studies on the human maternal brain is the implication of two networks that are frequently co-activated: the salience network and the emotion regulation network (Seeley et al. 2007). The *salience network* is activated in response to the most relevant stimuli resulting in a state of alert being fundamental, among others, for threat detection. Maternal concerns focus on the well-being of the infant where vigilant protectiveness and harm-avoidant behaviors are essential. This network is built around paralimbic structures—particularly the dorsal anterior cingulate and orbital frontoinsular cortices—and has a strong connectivity to subcortical and limbic structures (Seeley et al. 2007). With regard to *the emotion regulation network*, a common challenge for parents is to preserve their own regulated emotional state in front of threats or while caring for their immature and deregulated child. Children often arouse many emotions, since they express their own emotions intensely and without control. The mother, in order to help her infant to integrate his/her emotional experience, must first effectively regulate her own emotional arousal and coordinate it with thoughts, actions, and interactions (Rutherford et al. 2015). In doing so, prefrontal and cingulate control systems modulate activity in emotion systems (Ochsner et al. 2012; Rutherford et al. 2015). During fMRI tasks, these networks are active in front of own-baby versus other-baby stimuli (Leibenluft et al. 2004), particularly if the stimuli presents a negative emotional cue, such as cries or distress (Lorberbaum et al. 2002; Noriuchi et al. 2008; Swain et al. 2008). Furthermore, fMRI studies that employ own-children cries positively correlate a greater activity in frontal regions to a better quality of attachment (Laurent and Ablow 2012a) and to more sensitive behaviors with their infant (Musser et al. 2012), and reduced activation in the anterior cingulate cortex and medial prefrontal cortex have been associated with greater cortisol reactivity to stress in primiparous women (Laurent et al. 2011).

fMRI studies also strongly converge on the observation that new mothers exhibit an activation of the *empathy* and *ToM networks* (Gingnell et al. 2015; Leibenluft et al. 2004; Lenzi et al. 2009). These networks, which are specialized in social cognitive processing, encompass regions of the reward, salience, and emotion regulation network. The anterior insula and middle anterior cingulate cortex play a fundamental role in empathy (Lamm et al. 2011), the capacity of sharing another’s emotional state while being conscious that the other is the source of that emotion (Kanske et al. 2015). ToM, often also called “mentalizing” or “social intelligence,” is based on the assumption that we possess a theory of mind that allows us to have a reflective function concerning our mental states and to attribute mental states to others, accepting that others can have perspectives, beliefs, desires, and intentions different from one’s own (Premack and Woodruff 1978; Schaafsma et al. 2015). The neural network underlying ToM includes midline areas (medial prefrontal cortex, precuneus, and posterior cingulate cortex) anterior temporal lobes, temporo-parietal junction, and the inferior frontal gyri (Schurz et al. 2014). Through empathy and perspective taking, the mother can “feel” and infer what her baby, incapable to speak, is experiencing and can consequently offer accurate care. Interestingly, using quantitative analyses of overlap, Hoekzema et al. (2017) confirmed a remarkable similarity between the structural changes of pregnancy and the ToM network, and found that only networks recruited by ToM tasks displayed a greater-than-random overlap with the observed morphological brain changes. Furthermore, it was observed that the neural response of mothers to visual cues of their babies corresponded to various regions that lost GM volume across pregnancy, supporting the notion of a specialization of the brain—more specifically, in the neural network subserving social cognition—that plays a role in a mother’s response to her infant (Hoekzema et al. 2017).

A meta-analysis has integrated the results of eight fMRI studies assessing mothers while processing own versus unknown infant stimuli (a total of 144 mothers, 27 with depressive symptoms or a personality disorder) (Rocchetti et al. 2014). The results of this meta-analysis showed a greater activation in the bilateral insula extending to the inferior frontal gyrus, basal ganglia, and thalamus, confirming the implication of these regions in the maternal circuitry.

A few recent studies show that the neurobiology of motherhood represents a dynamic process: a longitudinal fMRI study examining mothers in the early postpartum period (within 48 h) and 1 month later using an emotional face-matching task, showed that the reactivity in the insula, middle frontal gyrus, and inferior frontal gyrus had incremented, suggesting that the neurobiology of motherhood continues to change throughout the postpartum period, perhaps in order to follow the constant development of the offspring (Gingnell et al. 2015). Parsons et al. (2017) revealed that as the child grows, the mother shows greater activity in response to general infant cues in key regions linked to caregiving, more specifically in the amygdala and orbitofrontal cortex, implicated in parental vigilance and the appraisal of emotional expressions. In other words, a lengthier maternal experience is associated with heightened, differential brain reactivity that reflects a buildup of these capacities over time. Montirosso et al. (2017) showed that giving birth to a preterm baby that needs neonatal intensive care is accompanied by a differential pattern of activity in areas subserving emotion regulation and social cognition (a greater activation in inferior frontal gyrus, supramarginal gyrus, and insula), reflecting that the neurobiology of motherhood is dynamic not only regarding the passage of time but also depending on the environmental circumstances, facilitating a sensitive adaptation to the infants’ needs.

Few studies have investigated brain activity during pregnancy, but they all indicate changes in the recruitment of neural resources in order to facilitate the processing of social information: Raz (2014) show changes in the recruitment of neural resources processing emotional stimuli, and Roos et al. (2011) found that PFC activation in front of fear-relevant stimuli is associated with increased distress and anxiety, and increased levels of cortisol and testosterone in pregnant but not in control women. Other studies that do not assess brain activity have found enhanced face recognition during pregnancy particularly for own-race male faces (Anderson and Rutherford 2011), enhanced ability to encode emotional faces of threat or harm during late pregnancy (Pearson et al. 2009) and an increase in ethnocentrism during the first trimester of pregnancy (Navarrete et al. 2007). In accordance with these findings, the notion of gestational adaptations in social cognition has been proposed from an evolutionary perspective (Anderson and Rutherford 2012), where enhanced social cognitive abilities and vigilance towards emotional signals would facilitate efficiently establishing alliances and identifying threats such as potential infections and aggressions from conspecifics in order to protect the fetus.

It is worth mentioning that, while pregnancy and the associated brain changes may help prepare a female for motherhood, this is not a requirement for mothering. For instance, virgin female rats will start to exhibit maternal behavior towards pups when exposed to pups consistently for a certain period (Seip and Morrell 2008). Alloparenting can also happen in females that are taking care of their offspring and adopt a parental role towards other unrelated infants (Schubert et al. 2009), as well as in males (Stiver and Alonzo 2011), and there are even cases of interspecies nursing. Among humans, even if mothers are often the primary caregiver, fathers, grandparents, other related kin and unrelated professionals can also take good care of and genuinely attach to a child. Likewise, the adoption of infants in humans illustrates that mother-child behavior and attachment can occur successfully without being preceded by endocrine priming. In fact, foster mothers show an association between oxytocin levels, caregiving behaviors, and brain activity in response to cues of the foster child (Bick et al. 2013); findings that are similar to those observed in biologically related mothers. Hence, pregnancy-related neuroendocrine changes cannot be necessary for—neither guarantee—the emergence of a dedicated mother taking care of and having a loving bond with her child.

In conclusion, it has been said that “it is fortunate for their survival that babies are so designed by Nature that they beguile and enslave mothers” (Bowlby 1958, p. 19). While it is true that babies are captivating, it appears that “nature,” in addition to its charming baby design, also designed the mother’s brain for this evolutionary goal.

## Peripartum mental disorders

Given the vast brain plasticity inherent to reproduction itself, it is unsurprising that the transition to motherhood is a delicate period accompanied by a higher risk of suffering mental disturbance. At least one in 10 new mothers finds herself with a surprising difficulty to take care of and enjoy her baby (Fisher et al. 2012). She may be facing a postpartum disorder which, if not treated, can have far-reaching consequences for both the mother and the child (Davalos et al. 2012). The possible severity of such consequences is demonstrated by records from the UK that indicate suicide as the leading cause of maternal death (Oates 2003).

Several types of mental problems can occur during the perinatal period. The most common is postpartum depression (PPD), with an estimated 11–20% of new mothers suffering from minor and approximately 7–14% from major depression (Almond 2009; Earls 2010; Gavin et al. 2005). Symptomatology is dysphoric affect, a disposition to feel guilty and/or ashamed, and impaired concentration. Another frequent complication of childbearing is anxiety. New mothers often experience some apprehension in response to childbearing, which is adaptive (Winnicott 1975), but some mothers experience a concern that is excessive and cannot be controlled, causing levels of distress and impairment. Anxiety disorders manifest in a wide range: generalized anxiety disorder, obsessive–compulsive disorder, panic disorder, and birth-related posttraumatic stress disorder. In the postpartum, sometimes the severity and symptoms do not rise to the level of an anxiety disorder diagnosis (e.g., hypervigilant concerns and attention for the baby, extreme lability, constant worry) but nevertheless can cause significant distress and disturb mother-infant interactions (Anniverno et al. 2013; Ross and McLean 2006). Rates of postpartum anxiety (PPA) range from 10 to 17% in primiparous women (Miller et al. 2006, 2013; Paul et al. 2013). PPD is frequently comorbid with PPA (O’Hara and Wisner 2014; Reck et al. 2008) and both mood disorders can also be manifested during pregnancy with an estimated prevalence of 15.6% (Fisher et al. 2012). In fact, a recent review estimates that rates of antenatal depression are higher than in the first year following childbirth (Underwood et al. 2016) and that both PPD and PPA are often a continuation of symptomatology already manifested antenatally (Heron et al. 2004).

Puerperal psychosis (PPP) has a low incidence, but evolves rapidly and can have tragic consequences. Several characteristics of its diagnosis are debated (Higgins 2012), but based on psychiatric income rates, it is estimated that the incidence ranges between 0.89 and 2.6 in 1000 births (VanderKruik et al. 2017), although others assure that the incidence is considerably higher and that the women who come to be admitted are only the most serious cases (Lucas 1994; Vesga-López et al. 2008). Sometimes, the woman already manifested anxiety or depression during pregnancy, but other times the psychosis arrives suddenly. The person begins to experience restlessness, insomnia, irritability, and, in many cases, euphoria. As the development is rapid, usually without prodrome, confusional states appear abruptly, where places, people, objects, and baby are perceived blurry, threatening, or bizarre. They are accompanied by strange behavior, lability of mood, and hallucinations and/or delusions about, for instance, the baby being dead, very sick, deformed, or possessed (Higgins 2012; Raphael-Leff 2014). There is an increased risk of filicide and suicide, usually carried out in an unforeseen way and with little planning (Brockington 2017).

As opposed to certain diseases with a clear biologically defined underlying pathology, peripartum mental disorders are determined by multiple factors and can therefore not be fully understood without taking into account multiple biological, psychological, and environmental influences, e.g., poor social support (Collins et al. 2011; Leigh and Milgrom 2008), stressful life events (Agrati and Lonstein 2016), a history of mental illness (Fisher et al. 2012), hormonal fluctuations (Altemus 2010; Seeman 1997), and a genetic predisposition (Agrati and Lonstein 2016; Caspi and Moffitt 2006). Therefore, eventually, an interdisciplinary approach is required to fully understand the etiology of peripartum mental disorders. Neuroscientific progress is considerably contributing to increasing our understanding of the neural mechanisms underlying these mental disorders, which is expected to lead to advances in prevention and treatment strategies.

### Brain structure in peripartum mental disorders

The neurobiology of peripartum mental disorders remains to be defined, but animal studies provide important indications. In laboratory rodent models, a repeated exposure to stressors, separation from pups, and the administration of hormones like glucocorticoids can produce PPD- and PPA-like behaviors. Furthermore, these models show brain alterations in neurogenesis and cellular morphology (e.g., reduced spine density, dendritic length, and branching) within key structures of the maternal circuitry such as nucleus accumbens, prefrontal cortex, bed nucleus of the stria terminalis, midbrain periaqueductal gray and hippocampus (Pawluski et al. 2017; Pereira 2016).

Few human studies have assessed structural alterations in puerperal clinical samples. A recent study utilized diffusion tensor imaging in order to compare white matter integrity between medication-free mothers with PPD and healthy control mothers (Silver et al. 2018). Region of interest analysis detected a white matter abnormality in the left anterior limb of the internal capsule in PPD, suggesting a disruption of fronto-subcortical circuits. In addition, correlation analyses relate disrupted interhemispheric structural connectivity to elevated depression scores (Silver et al. 2018). It is not clear if this disrupted structural connectivity represents a structural risk factor for the development of peripartum depression or is pathognomonic of a PPD episode, but interestingly, Young et al. (2017), based on a review of fMRI studies, conclude that there is an association between more adaptive maternal behavior and good regulation across prefrontal and subcortical regions.

Regarding PPP, a computerized tomography study compared mothers with PPP to patients (with psychoses or bipolar disorders outside the postpartum period) and controls (Lanczik et al. 1998). PPP mothers showed a volume enlargement in the left ventricular area, planimetric ventricular-to-brain ratio, and superior cerebellar cistern. Another study examined whether mothers who develop a PPP episode differ in brain cortical volume and morphology (Fusté et al. 2017). They compared three groups of mothers at the postpartum period: 11 mothers at risk that developed a PPP episode; 15 mothers at risk that did not develop a PPP episode; and 21 control mothers. Region of interest analyses showed that mothers with an episode had less volume in the ACC, left parahippocampal gyrus, and left superior temporal gyrus in comparison to mothers at risk without an episode. Subsequent analyses showed that these differences might be driven by surface area changes rather than changes in cortical thickness. In addition, mothers at risk without an episode had larger left superior and inferior frontal gyrus than the control group, proposing a different cortical phenotype across the three groups. Since there were between-group differences in terms of duration of illness and interval between delivery and the MRI acquisition, the results should be interpreted with caution. Nevertheless, they provide information about alterations on brain morphology in relation to PPP.

### Brain function in peripartum mental disorders

fMRI studies assessing samples with PPD or high levels of anxiety have identified a different pattern of neural activation in front of own-infant cry in comparison to control mothers, suggesting reduced neural sensitivity. For instance, mothers with PPD have shown weaker activation of reward and motivation areas such as the thalamus, nucleus accumbens, and caudate and key emotion regulation areas such as the lateral orbitofrontal cortex (Laurent and Ablow 2012b). Kim et al. (2015) found that higher levels of anxiety in relation to parenting correlated with reduced activation in the substantia nigra, a region implicated in reward processing.

Results appear to be contradictory in relation to the involvement of the amygdala: Some studies relate a more adaptive caregiving behavior to an increased activity while others relate this to a decreased activity (Barrett et al. 2012; Laurent and Ablow 2012a; Lenzi et al. 2016; Moses-Kolko et al. 2010; Wonch et al. 2016). In relation to this, Young et al. (2017) hypothesize that sensitive maternal care lies on a U-shaped curve, where both hypo-reactivity and hyper-reactivity to infant cues in the amygdala are problematic. Mothers suffering from PPD or PPA may fall at either end of this curve and therefore show disrupted maternal sensitivity.

Resting-state fMRI is another strategy that has been used to investigate brain activity in peripartum mental disorders. It explores spontaneous neural activity and allows investigating the so-called Default Mode Network. Interestingly, this network encompasses internally focused, self-referential processing (Buckner et al. 2008; Northoff et al. 2006; Wicker et al. 2003) as well as ToM functions (Mars et al. 2012; Uddin et al. 2007). This analogous involvement of the same midline structures in both self- and other-referential processing corresponds to the simulation theory that says that we use our own mental state as a model for inferring the mental states of others (Goldman 1992) and not by merely simulating being in the other’s situation but by simulating “being the other” with his or her psychological traits (Gordon 1995). ToM, as indicated above, is fundamental for a mother, since it enables her to make sense of the observed behavior of her baby. Three studies have shown abnormal neural activity in medication-free mothers with PPD when compared to HCM: Xiao-juan et al. (2011) showed that regional homogeneity was significantly increased in the posterior cingulate and medial frontal and decreased in temporal gyrus, Deligiannidis et al. (2013) showed that cortico-cortical and cortico-limbic connectivity was significantly blunted, and Chase et al. (2014) found a clear disruption of PCC connectivity with the right amygdala.

Interestingly, when comparing PPD and PPA to the same symptom profile at other times in life outside the postpartum period (major depressive disorder (MDD) or general anxiety disorder (GAD)), some neurobiological distinctions can be discerned: Pawluski et al. (2017) noted that resting-state activity and functional paradigms using emotional cues in individuals with MMD show a hyperactivity in medial affective and limbic regions that is not present in women with PPD. With regard to anxiety symptomatology, subjects with GAD show a hyperactivity in the amygdala and insula in response to emotional cues, as well as an increased connectivity between these two areas. On the other hand, higher anxiety in postpartum women is associated with a lower amygdala response to emotional non-own-infant cues and lower connectivity between the amygdala and insula (Pawluski et al. 2017). Better discerning the distinctions between these disorders occurring during and outside of the perinatal period is important, not only for increasing our understanding of the neurobiology of these disorders but also to aid therapeutic development, since a different neurobiological profile may require a distinct treatment approach.

In summation, pregnancy and the postpartum period differ significantly from other times in a woman’s life in terms of developing a de novo mental disorder or experiencing a relapse of a pre-existing disorder. The etiology of these disorders is multifactorial, but progress in neurobiological research is leading to significant clues. Animal models and neuroimaging studies in humans are starting to detect abnormalities in structure, function, and connectivity in brain regions responsible for ToM, self-regulation, and emotion in mothers with perinatal disorders. Although there are similarities between mood and psychotic disorders during the peripartum period and those occurring in other times in life, significant differences are also starting to emerge (Fusté et al. 2017; Pawluski et al. 2017), which might eventually lead to improvements in the treatment and prevention of these disorders.

## Conclusion

A compelling body of evidence in healthy women and other female mammals confirms that, during pregnancy and the postpartum period, hormones and sensory interactions with the offspring relate to complex structural and functional changes in the brain. This reproduction-related brain plasticity embraces various areas implicated in maternal caregiving, primarily regions involved in reward/motivation, salience/threat detection, emotional regulation, and social cognition such as the ability to empathize and infer the mental state of the baby. These changes seem necessary and adaptive: since the survival of the young is dependent on the mother’s efforts, her brain seems to have evolved in ways that promote mother-infant bonding and sensitive caregiving.

Although this maternal brain plasticity facilitates a higher purpose—the continuation of the species—it is not necessarily innocuous and predisposes the mother or mother-to-be to peripartum mental disorders. Irrespective of the clinical manifestation of the disturbance, the suffering that accompanies a peripartum disorder has important implications for the mother, her partner, and her child. As briefly reviewed here, brain research is offering growing scientific understanding concerning the neural correlates of these disorders. Some structural irregularities and differences in activation patterns are starting to be identified, which will eventually lead to improvements in prevention and treatment.

Since pregnancy and the postpartum period signify a period of particular vulnerability for the mother as well as for the newborn—who is highly susceptible to his/her mother’s distress or well-being—the antenatal period can be seen as a unique opportunity to identify women at risk. Just as we take care of the women’s physical health during gestation and the peripartum period—with programmed obstetric visits and skilled birth attendance, safeguarding many lives—taking care of a woman’s mental health during this time seems a promising focus for scientific and clinical investment.
